# Metabolic engineering of *Halomonas campaniensis* strain XH26 to remove competing pathways to enhance ectoine production

**DOI:** 10.1038/s41598-023-36975-8

**Published:** 2023-06-15

**Authors:** Zhiwan Shu, Xin Zhang, Rong Wang, Jiangwa Xing, Yongzhen Li, Derui Zhu, Guoping Shen

**Affiliations:** Research Center of Basic Medical Science, Medical College of Qinghai University, Xining, 810016 China

**Keywords:** Microbiology, Molecular biology

## Abstract

Ectoine has gained considerable attention as a high-value chemical with significant application potential and market demand. This study aimed to increase ectoine yields by blocking the metabolic shunt pathway of l-aspartate-4-semialdehyde, the precursor substrate in ectoine synthesis. The homoserine dehydrogenase encoded by *hom* in *H. campaniensis* strain XH26 is responsible for the metabolic shunt of l-aspartate-4-semialdehyde to glycine. CRISPR/Cas9 technology was used to seamlessly knockout *hom*, blocking the metabolic shunt pathway to increase ectoine yields. The ectoine yield of XH26/*Δhom* was 351.13 mg (g CDW)^−1^ after 48 h of incubation in 500 mL shake flasks using optimal medium with 1.5 mol L^−1^ NaCl, which was significantly higher than the 239.18 mg (g CDW)^−1^ of the wild-type strain. Additionally, the absence of the ectoine metabolic shunt pathway affects betaine synthesis, and thus the betaine yields of XH26/*Δhom* was 19.98 mg (g CDW)^−1^, considerably lower than the 69.58 mg (g CDW)^−1^ of the wild-type strain. Batch fermentation parameters were optimized, and the wild-type strain and XH26*/Δhom* were fermented in 3 L fermenters, resulting in a high ectoine yield of 587.09 mg (g CDW)^−1^ for the defective strain, which was significantly greater than the ectoine yield of 385.03 mg (g CDW)^−1^ of the wild-type strain. This study showed that blocking the metabolic shunt of synthetic substrates effectively increases ectoine production, and a reduction in the competitively compatible solute betaine appears to promote increased ectoine synthesis.

## Introduction

Ectoine is a derivative of amino acid, 1,4,5,6-tetrahydro-2-methyl-4-pyrimidnecarboxylic acid, that is found intracellularly in a diverse range of organisms such as halophilic archaea, bacteria, fungi, and algae^1–4^. Its ability to enhance enzymatic activities, stabilize DNA structures, and protect proteins improves salt-stress resistance, making ectoine a valuable compound in various applications^5–8^. Additionally, ectoine acts as a free radical scavenger, and its anti-inflammatory properties have been utilized in biomedical applications, including skin trauma, allergic rhinitis, dry eye, and lung and intestinal diseases^9–14^. Therefore, due to its widespread application potential and market demand, ectoine is considered a promising high-value chemical. However, the chiral carbon atom structure of ectoine makes chemical synthesis challenging. Thus, microbial fermentation, which is efficient and cost-effective, is the primary large-scale source of ectoine^15,16^.

The metabolism of l-aspartate-4-semialdehyde, a forerunner substrate in the ectoine biosynthesis pathway, relies on enzymes encoded by the conserved gene clusters *ectABC* or *ectABCD-ask*^17^. The produced metabolite then undergoes a three-step enzymatic reaction to produce ectoine. In this pathway, the *ectB*-encoded diaminobutyric acid-2-oxoglutarate transaminase (EctB) first converts l-aspartate-*β*-galacturonide to l-2, 4-diaminobutyric acid (DABA). Second, DABA is converted to *N*-acetyl diaminobutyric acid (NADA) by the *ectA*-encoded l-2, 4-diaminobutyric acid aminotransferase. Finally, NADA is converted to ectoine by ectoine synthase, which is encoded by *ectC*^18–20^. Wild-type bacterial strains, which are typically obtained via direct screening surveys from natural environments, have a limited capacity for the intracellular accumulation of ectoine and cannot meet the needs of practical production. In addition, the high cost of producing ectoine from wild-type strains renders large-scale ectoine production difficult. Thus, ectoine overproduction has become an important area of research, and previous studies have identified excellent industrial strains, modified overproducing strains, constructed genetically engineered strains, and/or systematically constructed metabolically engineered strains that can be effectively used for ectoine overproduction^21–25^. Metabolically engineered strains with high ectoine production can be systematically generated using gene knockout technology targeting the ectoine biosynthesis metabolic pathway. For example, disrupting the glyoxalate cycle by knocking out its control gene *iclR* increases metabolic flow from the carbon source glucose to L-aspartic acid, generating strains with high ectoine yields (i.e., ectoine yields of 12–18 g ectoine L^−1^ after 6–8 h of fractionated fermentation)^26^. Similarly, knockout of the genes encoding the aspartate kinase/homoserine dehydrogenase *thrA* and the glyoxylate shunt transcriptional repressor gene *iclR* increases the oxaloacetate pool to produce strains with high ectoine yields (i.e., ectoine yields of 25.1 g ectoine L^−1^ with glucose fermentation under low salt conditions)^27^. It is equally possible to achieve high ectoine yields by knocking out key or nodal genes in the metabolic shunt pathways to disrupt competing pathways^28^.

**Figure 1:**
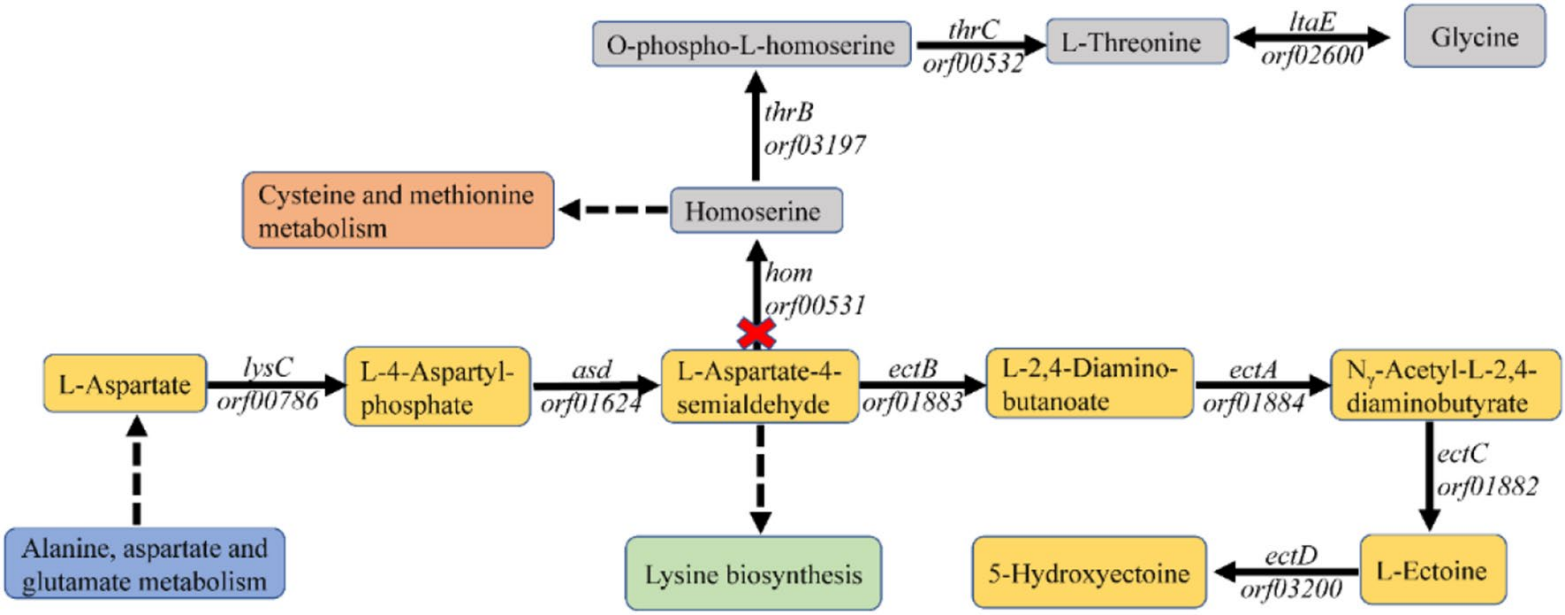
Hypothesized effects of *hom* knock out on the ectoine biosynthesis pathway of *Halomonas* strain XH26. *lysC*: aspartate kinase; *asd*: aspartate-semialdehyde dehydrogenase; *ectB*: diaminobutyrate-2-oxoglutarate transaminase; *ectA*: L-2,4-diaminobutyric acid acetyltransferase; *ectC*: L-ectoine synthase; *ectD*: ectoine hydroxylase; *hom*: homoserine dehydrogenase; *thrB*: homoserine kinase; *thrC*: threonine synthase; *ltaE*: threonine aldolase. The dashed lines represent substances exported or imported by other metabolic pathways. The colored boxes correspond to metabolic pathways.

The synthesis and accumulation of ectoine, a compatible solute, has been established as a significant process in various halophilic bacteria, including *Halomonas*. Ectoine is an amino acid derivative whose synthetic precursors are closely related to Asp and l-aspartate-4-semialdehyde. The latter is a crucial precursor in the biosynthesis of three distinct substances: ectoine, l-lysine, and glycine. The *H. campaniensis* strain XH26 is known to thrive in a wide range of salinities, from 0 to 3 mol L^−1^ NaCl, and produces multiple compatible solutes, exhibiting high ectoine yields^29^. Notably, *hom*-encoded homoserine dehydrogenase catalyzes the metabolic shunt of l-aspartate-4-semialdehyde to glycine, and knockout of *hom* is expected to increase ectoine synthesis effectively (Fig. 1). In this investigation, we employed a CRISPR/Cas9 system to knock out *hom* in the *Halomonas campaniensis* strain XH26, thus preventing the transformation of l-aspartate-4-semialdehyde into glycine, and examined the resulting impact on ectoine yields.

## Materials and methods

### Bacterial strains, plasmids, and culture conditions

All bacterial strains and plasmids used in this study are shown in Table 1. Wild-type *H. campaniensis* strain XH26 (CCTCCM2019776) was isolated from the Xiaochaidan Salt Lake (Qinghai Province, China) and deposited in the China Center for Type Culture Collection (CCTCCM, Wuhan, China). *E. coli* DH5α and *E. coli* Stab13 cells were used as hosts for molecular cloning. The donor vector plasmid pMD19-T-L + R was used to seamlessly repair DNA sequences after knockout to prevent insertion and deletion mutations. The basal culture medium is composed of NaCl 50 g L^−1^ (adjustable), MgSO_4_·H_2_O 25 g L^−1^, KCl 55 g L^−1^, CaCl_2_ 0.2 g L^−1^, sodium citrate 3 g L^−1^, sodium l-glutamate 6.5 g L^−1^, enzymatically hydrolysed casein 7.5 g L^−1^ and Yeast Extract 2 g L^−1^.

**Table 1:** Bacterial strains and plasmids used in this study.

Strains and plasmids	Functional characteristics	Source
Strains		
* H. campaniensis* strain XH26	Wild type	Laboratory stock, deposited at CCTCCM, China
* E. coli* DH5*α*	Cloning host	Purchased from TaKaRa Bio. Inc. (Dalian, China)
* E. coli* Stab13	Cloning host	Purchased from Sangon Bio. Inc. (Shanghai, China)
Strain XH26/*Δhom*	Strain XH26 that is *hom* deficient	This study
* E. coli* DH5α/pUC57-sgRNA	*E. coli* DH5α containing recombinant plasmid pUC57-sgRNA (2879 bp)	This study
* E. coli* DH5α/pMD19*-*T-L	*E. coli* DH5α containing recombinant plasmid pMD19-T-L	This study
* E. coli* DH5α/pMD19*-*T-R	*E. coli* DH5α containing recombinant plasmid pMD19-T-R	This study
* E. coli* Stab13/pwtCas9-bacteria	*E. coli* Stab13 containing plasmid pwtCas9-bacteria	This study
* E. coli* DH5α/pMD19-T-L + R	*E. coli* DH5α containing recombinant plasmid pMD19-T-L + R	This study
Plasmids		
pMD19*-*T	*Lac* promoter, cloning vector, Amp^r^	Purchased from TaKaRa Bio Inc (Dalian, China)
pUC57	*Lac* promoter, cloning vector, Kan^r^	Purchased from Genscript Bio Inc (Nanjing, China)
pwtCas9-bacteria	*TetR*/*tetA* promoter, *AmpR* promoter, bacterial knockout vector, Amp^r^, Tet^r^	Purchased from Miaoling Bio Inc (Wuhan, China)
pUC57-sgRNA	pUC57 containing the sgRNA	This study
pMD19-T-L	pMD19*-*T containing the left arm of the donor vector	This study
pMD19-T-R	pMD19*-*T containing the right arm of the donor vector	This study
pMD19-T-L + R	pMD19*-*T containing the donor vectors (L + R)	This study

### Genome analysis of ectoine metabolite pathway of *H. campaniensis* strain XH26

Based on the whole genome sequence of *H. campaniensis* strain XH26(NCBI no: CP071889)^29^, the coding sequences of the associated genes and transcriptional regulators involved in ectoine metabolism were mined and validated using BLAST searches against NCBI database. The ectoine metabolic pathway of *H. campaniensis* XH26 was evaluated using KEGG (Kyoto Encyclopedia of Genes and Genomes) database pathway analysis and salt-induced (0 M NaCl, 1.5 M NaCl, and 2.5 M NaCl) transcriptomic profiling^30,31^.

### Construction of knockout strains

To completely knockout the entire *hom* gene, the target gene site was designed and sgRNA fragments were identified using the CRISPOR (http://crispor.tefor.net/) or CRISPRdirect (http://crispr.dbcls.jp) platforms. The sgRNA sequence (5′-GGTATCACGGGCAACATCCAGCAGG-3′) was synthesized and cloned into the pUC57 vector to create the recombinant plasmid pUC57-sgRNA^32^. Genomic DNA was extracted from *H. campaniensis* strain XH26 using the Bacteria Genomic DNA Extraction Kit (9763, Takara Bio Co., China). The overlapping RCR amplification was performed to construct the fulfilment donor vector (plasmid pMD19-T-L + R) and generate the defective gene fragment, using specific primers listed in Table 2 and genomic DNA as template. The donor vector, lacking 1347 bp in *hom*, was ligated into the pMD19-T and transferred into the strain *E. coli* DH5α^33,34^. The Wild-type strain XH26 was inoculated into liquid media formulated for strain activation. After activation, the cells were inoculated into 50 mL of liquid medium and incubated at 37 °C until reaching OD_600_ values of 0.5–0.8. Cells were subsequently incubated in an ice bath for 10 min and collected by centrifugation at 6000×*g* for 7 min at 4 °C. Collected cells were washed with pre-cooled MgCl_2_ and PEG, followed by a final resuspension in PEG^35,36^. The plasmids pMD19-T-L + R, pUC57-sgRNA, and pwtCas9-bacteria were extracted from *E. coil* DH5α/L + R-T, *E. coli* DH5α/pUC57-sgRNA, and *E. coli* Stab13/pwtCas9-bacteria cells, respectively, using a plasmid extraction kit (9760, Takara Bio Co., Ltd., China). The successfully transformed cells were incubated at 37 °C for 2 h. The pMD19-T-L + R, pUC57-sgRNA, and pwtCas9-bacteria plasmids were transformed into strain XH26 in batches and screened using a solid plate medium containing ampicillin and tetracycline (T8180, Solarbio Life Science Inc., Beijing, China). Monoclonal strains were selected and verified by PCR amplification and DNA sequencing.

**Table 2:** The primers used in this study.

Primer name	Sequence (5′–3′)
*hom*-L-arm-F	CACGCATTTGCGACTCATTT
*hom*-L-arm-R	AGGTGCCGCCTCTTAAATCAG
*hom*-R-arm-F	CTCATGCGTTATATCAGCACG
*hom*-R-arm-R	CTGTTTGATGCTTACGACC
overlap PCR-F	ATAGCGCTGAGACACTGATTTAAGAGGCGGCACCTCTCATGCGTTATATCAGCACG
overlap PCR-R	GCGGGCGCTTGGCCACGCGTGCTGATATAACGCATGAGAGGTGCCGCCTCTTAAATCAG
*ectA*-F	CAGTCGCTGATGCTGTGGTTGG
*ectA*-R	GAATTAACATCAAGCGGCGGACAAG
*ectB*-F	TGCGTGGTATTGATGTTGTCTCTGG
*ectB*-R	CACTTCACTACTTCGCCGTCTTGG
*ectC*-F	GCTATGAAGGCGAAGGCGAAGTAG
*ectC*-R	AACAGATGTTCGTCGTGCTGATCC

### Genes related to ectoine synthesis in *H. campaniensis* strain XH26

In this study, total bacterial RNA was extracted from *H. campaniensis* strain XH26 using Trizol base lysis, under different salt gradient conditions [NS: no salt (0 mol L^−1^ NaCl), MS: medium salt (1.5 mol L^−1^ NaCl) and HS: high salt (2.5 mol L^−1^ NaCl)] and in triplicate. The purity of the extracted RNA was evaluated using a Nanodrop 2000 spectrophotometer (ND200, Thermo Fisher Scientific, USA), while RNA concentration was measured using a Qubit 2.0 Fluorometer (Q32867, invitrogen, USA). The cDNA was synthesized using a reverse transcription kit as per the manufacturer's instructions and the resulting reverse transcription products were stored at − 20 °C. Real-time quantitative PCR was performed to assess gene expression in response to different salt gradient conditions. The qRT-PCR primers were designed using the Primer 5.0 software and the primer sequences are shown in Table 2. Polymerase reactions were performed according to the qRT-PCR kit operation and run on a machine (Roche Light Cycler^®^ 480 II, Roche, Switzerland) with run parameters of 95 °C for 3 min; 95 °C for 10 s, 65 °C for 20 s and 72 °C for 30 s for 40 cycles. The internal reference gene used was *GADPH* and each biological sample was analysed in triplicate. The relative expression levels of each gene were calculated using the 2^−ΔΔCT^ method^37^.

### Determination of ectoine and betaine yields

To determine ectoine and betaine production yields, strains XH26 and XH26/*Δhom* were cultivated in tubes with basal culture medium (with 5 g L^−1^ glucose supplementation) for seed preparation, by incubated at 35 °C, 150 rpm until OD_600_ = 0.6. With same biomass inoculation, these seed culture was inoculated to 500-mL baffled flask containing 250 mL of NaCl-gradient (0, 0.5, 1.0, 1.5, 2.0, 2.5, 3.0 mol L^−1^) basal culture medium supplemented with 5 g L^−1^ glucose. After shaking incubation at 35 °C for 48 h (ZQTY-90S, Zhichu Instrument CO., Ltd., China), cultures were sampled for measuring the cell growth and ectoine production. Three independent experiments were conducted to avoid artifacts caused by handling.

### Determination of ectoine production from batch fermentation

The pre-cultured cells were inoculated into a 3 L microbial fermenter (MBF300ME, Tokyo RIKEN Co., Ltd, Japan) at 35 °C for batch fermentation. The yeast extract in the XH26 basal culture medium (with 5 g L^−1^ glucose supplementation) was then replaced by 0.5% aspartic acid as a nitrogen source. During fermentation, dissolved oxygen was controlled at 40% for 0–24 h and 20% for 24–72 h by means of an auto-adjusted agitation from 80 to 400 rpm, with the working volume of 1.8 L in the 3 L bioreactorand and a salinity control of 1.5 mol NaCl L^−1^^38–41^. The pH of the fermentation condition of cells was controlled at pH 8.0 with addition of appropriate amounts of 0.1 N NaOH and 0.1 N HCl. Cofactors such as biotin (0.3 µg mL^−1^) can regulate the microbial cell metabolism, and thus promoted the metabolism of amino acids and enzyme synthesis^42^. Therefore, appropriate amounts of biotin were added to the fermentation process to increase the overall production of ectoine.

### Analytical methods

Cell growth was monitored based on the optical density of the sample at the wavelength of 600 nm was measured with the UV/Visible spectrophotometer (SP-754, Shanghai Spectrum Instruments Co., Ltd., China). Ectoine and betaine concentrations in the extracts were determined using high-performance liquid chromatography (HPLC) with an ectoine standard curve as a reference^43^ as follows: First, 1 mL of each bacterial culture was centrifuged at 8000×*g* for 5 min and the supernatant was discarded. Then, 1 mL of ultrapure water was added to each tube, and the tube contents were ground for 5 min using a third-generation variable speed TGrinder (OSE-Y50, Tiangen Biotech Co., Ltd., China). After centrifugation at 8000×*g* for 5 min, the water phase (containing compatible solutes) was separated and filtered through a 0.22 µm filter membrane for HPLC analysis. Ectoine was identified using an Agilent Technologies 1260 Infinity HPLC instrument (USA) with a SeQuant ZIC-HILIC column (15.0 cm by 4.6 mm; 5 μm) (Sigma-Aldrich, USA). A solution of acetonitrile (1000161000, Sigma-Aldrich, USA) and pure water (v/v: 80/20) was used as the mobile phase, and the instrument was operated at a detection wavelength of 210 nm, a flow rate of 1.0 mL min^−1^, a column pressure of 3.486–4.761 MPa, a column temperature of 30 °C, and a sample volume of 10 µL. Betaine detection condition was as follows: a solution of acetonitrile and pure water (v/v: 85/15) was used as the mobile phase, detection wavelength of 195 nm, fow rate of 0.7 mL min^−1^, column pressure of 3.486–4.761 MPa, column temperature of 30 °C, and sample volume of 15 μL. A standard curve was generated using gradient-diluted compatible solute standards.

## Results

### Integrated reconfiguration of the ectoine metabolic pathway in *H. campaniensis* strain XH26

**Figure 2:**
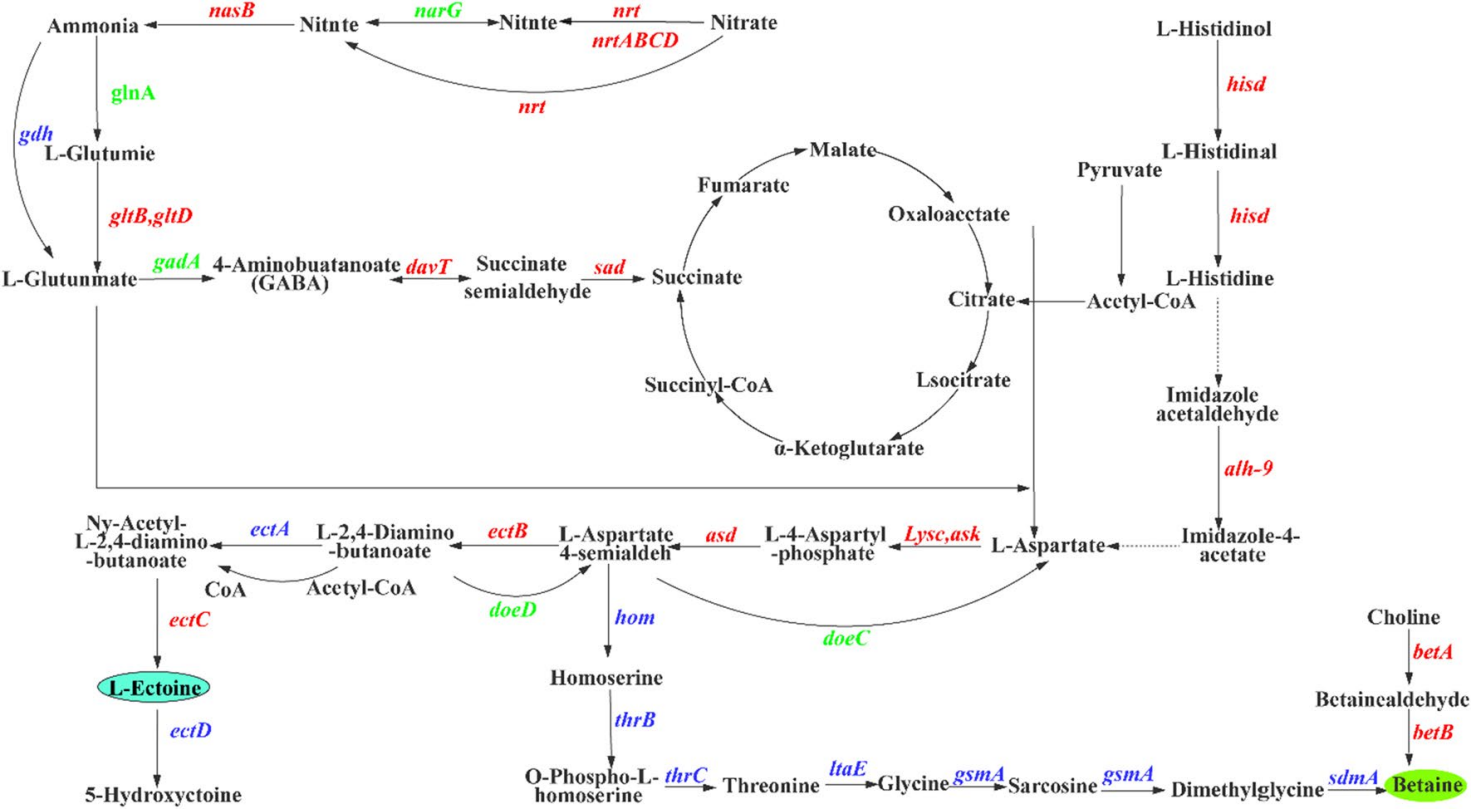
Diagram of the ectoine metabolism-related pathway of *H. campaniensis* XH26 in this study. The ectoine metabolism-related pathway of *H. campaniensis* XH26 was reconstructed based on salt-induced transcriptome analysis.

The synthesis and accumulation of compatible solute ectoine is a crucial adaptation mechanism in many halophilic bacteria, such as *Halomonas*, including *H. elongata* DSM 2581^T^, *H. hydrothermalis* Y2^30,44^. Analysis of high-throughput sequencing data, including genomic, salt-excited transcriptomic, and proteomic data from *H. campaniensis* strain XH26, identified multiple genes associated with ectoine anabolism along various metabolic pathways. Asp, oxaloacetate and Glu were used as metabolic nodes to screen for differentially expressed genes or related transcription factors involved in metabolism pathways directly or indirectly associated with ectoine metabolic (Table 3). KEGG analysis revealed that ectoine biosynthesis is associated with Asp, aspartate hemiacetal metabolism, and these metabolic pathways are in turn linked to aspartate (Asn), glutamate (Glu), glutamine (Gln) and alanine (Ala). At the same time, the carbon/nitrogen metabolic flow is associated with the tricarboxylic acid cycle (α-ketoglutarate, succinic acid, ferredoxin, malic acid, oxaloacetate) and amino acid (Glu and Asp) transamination (Fig. 2)^45–47^. In the NS/MS comparison group, transcript expression of genes *lysC*, *ectB*, *ectA*, *ectC*, *gltB*, *gltD*, *davT*, *hisD*, *alh-9*, *betA*, *acnB*, *pckA* and *gadA* was up-regulated, while transcript expression of genes *asd* and *gdhA* was down-regulated. In the NS/HS and MS/HS comparator groups, the genes *lysC*, *asd*, *ectB*, *ectC*, *gltB*, *gltD*, *davT*, *hisD*, *alh-9* and the gene *betA* were up-regulated and the genes *ectA*, *acnB*, *pckA*, *gadA* and *gdhA* were down-regulated. At the level of protein translation, there were no significant differences in the translation levels of most of the proteins, except for AcnB and EctB. The expression levels of AcnB and *acnB* followed the same trend, i.e. increasing and then decreasing with increasing salinity; the expression levels of EctB and *ectB* also followed the same trend (increasing with increasing salinity). Proteins act as executors of gene functions, and most gene protein translation levels in the ectoine synthesis pathway do not change significantly with increasing salinity, but the fact that ectoine production is increased is undeniable, and therefore increased protein EctB expression, may be the main cause of increased ectoine production. EctB is the key enzyme that catalyzes the change of l-aspartate-4-semialdehyde to l-2.4-Diamino-butanate, indicating that this pathway plays a key role in the synthesis of ectoine. Increasing the level of the enzyme in the synthetic pathway can effectively increase the yield of ectoine, and on this basis, increasing the concentration of the protein EctB catalytic substrate l-aspartate-4-semialdehyde in this pathway by knocking out *hom* can also further effectively increase the yield of ectoine.

**Table 3:** Key genes and transcriptional regulators associated with ectoine metabolism pathway.

Gene id	Gene name	Description of genes or factors	Regulate
*orf00786*	*lysC*	Aspartate kinase	Up
*orf01624*	*asd*	Aspartate-semialdehyde dehydrogenase	Down/up
*orf01883*	*ectB*	Diaminobutyrate-2-oxoglutarate transaminase	Up
*orf01884*	*ectA*	Diaminobutyrate acetyltransferase	Up/down
*orf01882*	*ectC*	l-ectoine synthase	Up
*orf03200*	*ectD*	Ectoine hydroxylase	Up
*orf02985*	*doeA*	Ectoine hydrolase DoeA	Up
*orf01061*	*doeB*	Succinylglutamate desuccinylase	Up/down
*orf02976*	*doeC*	NAD-dependent succinate-semialdehyde dehydrogenase	Down
*orf02975*	*doeD*	Aspartate aminotransferase family protein	Down
*orf00775*	*gltB*	Glutamate synthase large subunit	Up
*orf00776*	*gltD*	Glutamate synthase	Up
*orf03510*	*alh-9*	Aldehyde dehydrogenase family protein	Up
*orf00758/orf02393*	*davT*	4-Aminobutyrate transaminase	Up
*orf02075*	*betA*	Succinate-semialdehyde dehydrogenase	Up
*orf02271*	*hisD*	Histidinol dehydrogenase	Up
*orf00874/ orf02411*	*gdhA*	Glutamate dehydrogenase	Down
*orf01682*	*acnB*	Aconitate hydratase B	Up/down
*orf00161/orf02497*	*pckA*	Phosphoenolpyruvate carboxykinase	Up/down
*orf03123*	*gadA*	Glutamate decarboxylase	Up/down
*orf01032*	*ohrR*	*marR* family transcriptional regulator	Up/down
*orf02730*	*pecS*	*marR* family transcriptional regulator	Down/up
*orf02502/orf03560*	*ydgJ*	*marR* family transcriptional regulator	Up/down

### Transcript level changes of ectoine metabolite related genes

**Figure 3:**
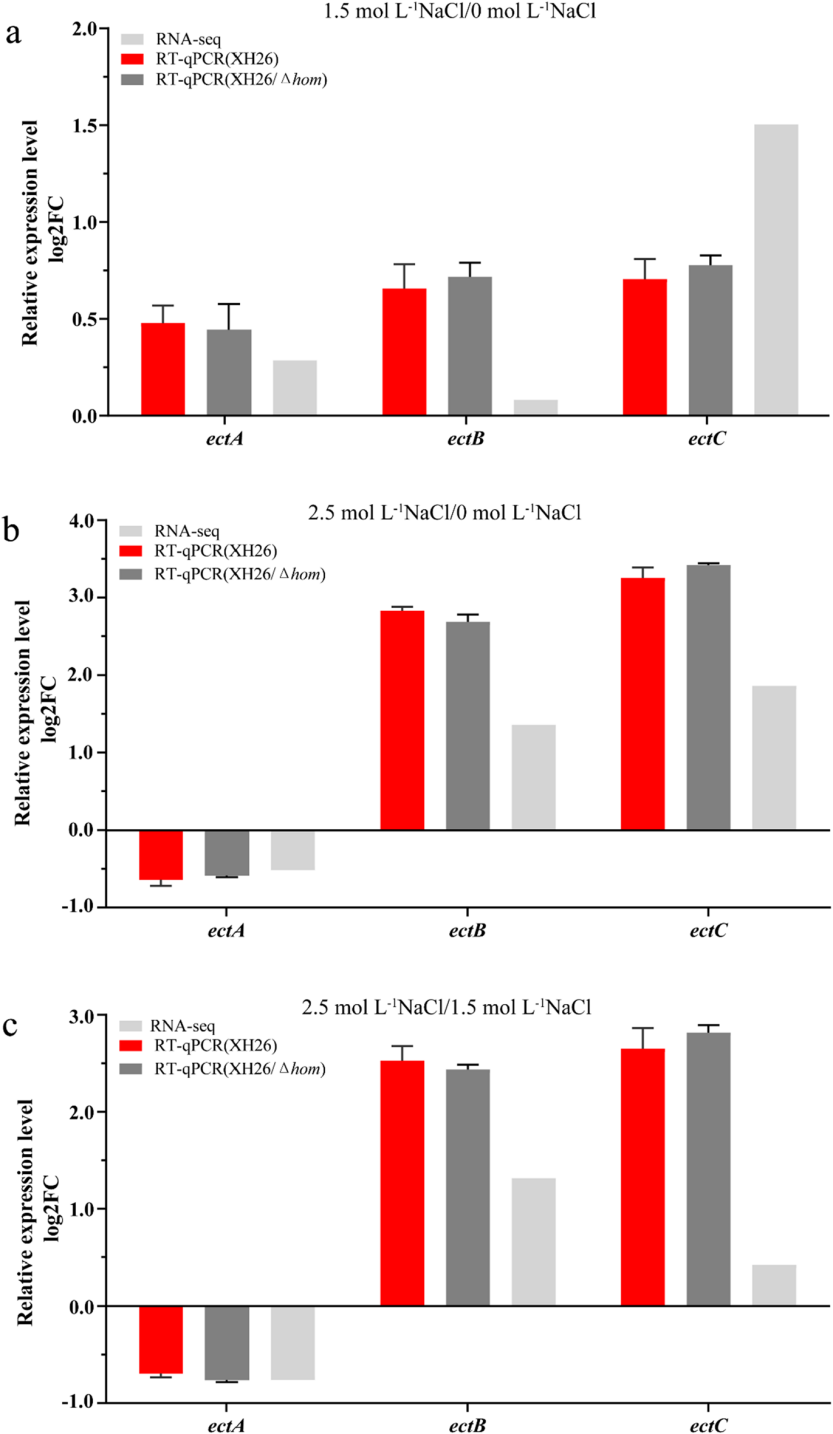
Mapping of differential expression levels of genes related to ectoine synthesis. RNA-seq and qRT-PCR results showing the expression changes of ectoine synthesis under salt stress. For qRT-PCR, data are presented as the mean ± standard error (n > 3).

The biosynthesis of ectoine relies on the *ectABC* or *ectABCD-ask* gene clusters, which are evolutionarily conserved^48,49^. These gene clusters are present in the genome of *H. campaniensis* strain XH26, with the *ectABC* cluster located at 19,453,512–1,945,966 in the genome. To verify transcriptional changes in the ectoine synthesis genes identified in RNA-seq, qRT-PCR was conducted to measure mRNA expression levels. No significant differences in transcript levels were observed in the *ectABC*-linked gene cluster between strain XH26 and strain XH26/*Δhom* (Fig. 3). In the HS/NS and HS/MS comparison groups, transcription of *ectB* and *ectC* was significantly up-regulated while transcription of *ectA* was down-regulated (p < 0.05, |log2FC|≥ 1). In the MS/NS comparison group, the transcript levels of ectoine synthesis genes were up-regulated in *ectA*, *ectB* and *ectC* expression (p < 0.05, |log2FC|≥ 1). High salinity stress resulted in reduced ectoine synthesis that correlated with reduced transcript levels of *ectA*. We found that knockout of gene *hom* seems to increase only the substrate concentration in the enzymatic reaction of ectoine synthesis and had no effect on the enzyme content.

### Ectoine and betaine fermentation capacity of the genetically defective strains

**Figure 4:**
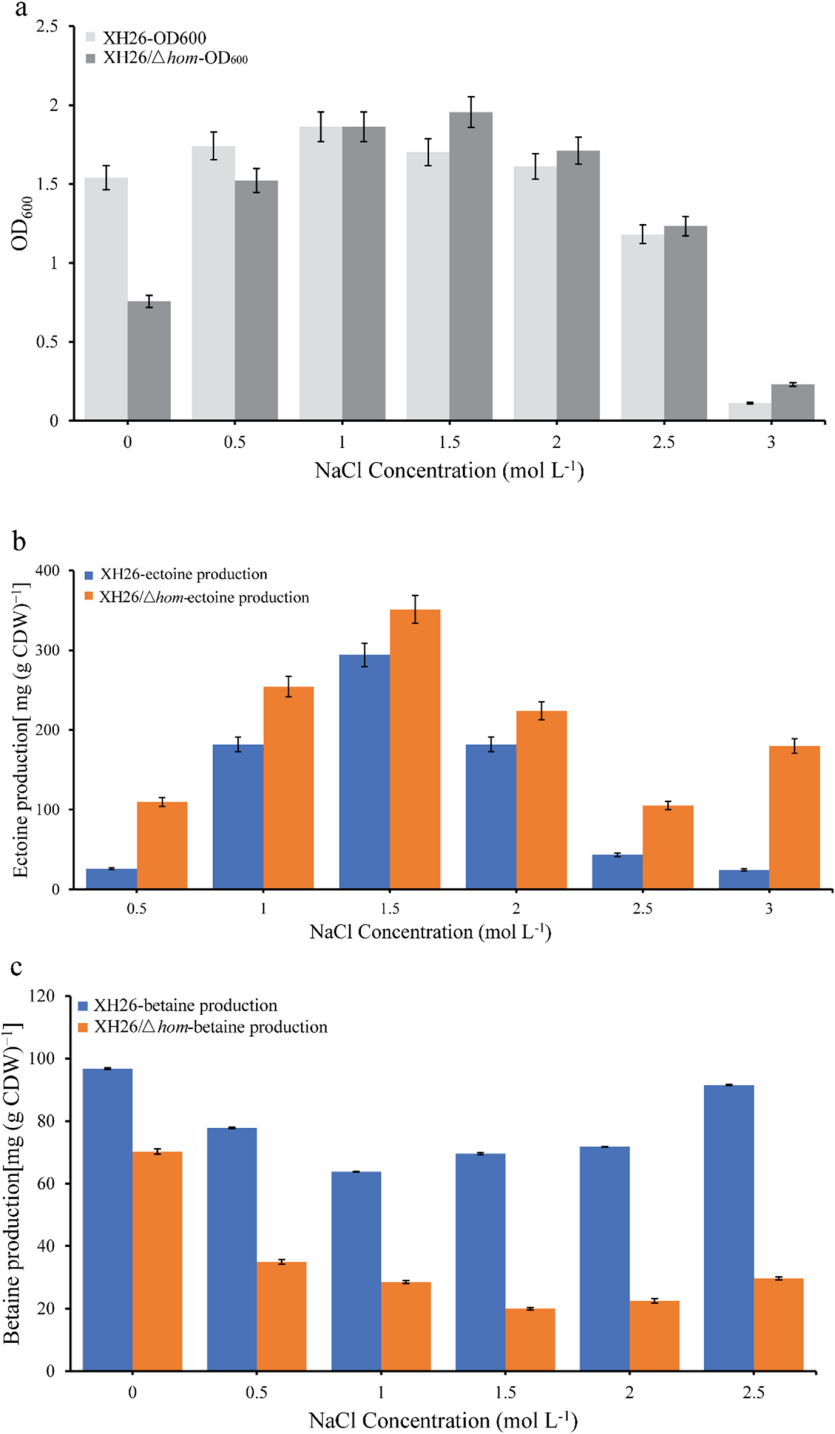
Growth (**a**), ectoine accumulation (**b**) and betaine accumulation (**c**) of the wild-type strain XH26 and the defective strain XH26/*Δhom* under various salinity conditions. Cultures were grown in 500 mL baffled Erlenmeyer flasks, and each experiment was performed in triplicate.

To investigate the role of *hom* in ectoine production, the entire *hom* gene was completely knocked out in the constructed strain XH26/*Δhom*. The growth and ectoine accumulation of the wild-type strain XH26 and the defective strain XH26/*Δhom* were compared after 48 h of incubation at various salinities (Fig. 4). The results showed that the maximum growth of XH26/*Δhom* was higher than that of the wild-type strain, and the salinity tolerance associated with maximum growth was also altered. The wild-type strain exhibited maximum growth at a salinity of 1 mol NaCl L^−1^ (OD_600_ value of 1.86), while the defective strain exhibited maximum growth at a salinity of 1.5 mol NaCl L^−1^ (OD_600_ value of 1.96) (Fig. 4a). Ectoine accumulation trends were generally similar between the two strains, although ectoine production was higher in the defective strain than in the wild-type strain at all salinities. The highest yield of ectoine [351.13 mg (g CDW)^−1^ of isoflavones L^−1^ in shake flask fermentation] was obtained by XH26/*Δhom* at a salinity of 1.5 mol NaCl L^−1^, which was 47% higher than the wild-type strain (Fig. 4b). *Halomonas* species contain diverse compatible solutes and use various substances to counteract salt stress, with betaine functioning under low salt conditions. Betaine fermentation experiments showed that the betaine yields of XH26/*Δhom* were lower than those of the wild-type strain under low salt conditions. In particular, the betaine production of XH26/*Δhom* was 19.98 mg (g CDW)^−1^ at 1.5 mol L^−1^ NaCl, much lower than the 69.58 mg (g CDW)^−1^ of the wild-type strain (Fig. 4c). At a salinity of 3.0 mol NaCl L^−1^, the ectoine production of the wild-type strain was 0 and the ectoine production of the defective strain remained relatively high, while the betaine production of both was 0. Whether the deficiency of betaine is responsible for the relative boost in ectoine yields and the link between them needs to be explored in considerable depth. Together, our experimental results showed that *hom* is a nodal gene in the ectoine synthesis pathway. Knockout of *hom* blocked the use of the precursor substrate (l-aspartate-4-semialdehyde) in the glycine biosynthesis pathway, thereby allowing more l-aspartate-4-semialdehyde to be used for ectoine synthesis and leading to increased ectoine production, consistent with the results of anticipated experiments.

### Batch fermentation of cells ectoine using bioreactor

**Figure 5:**
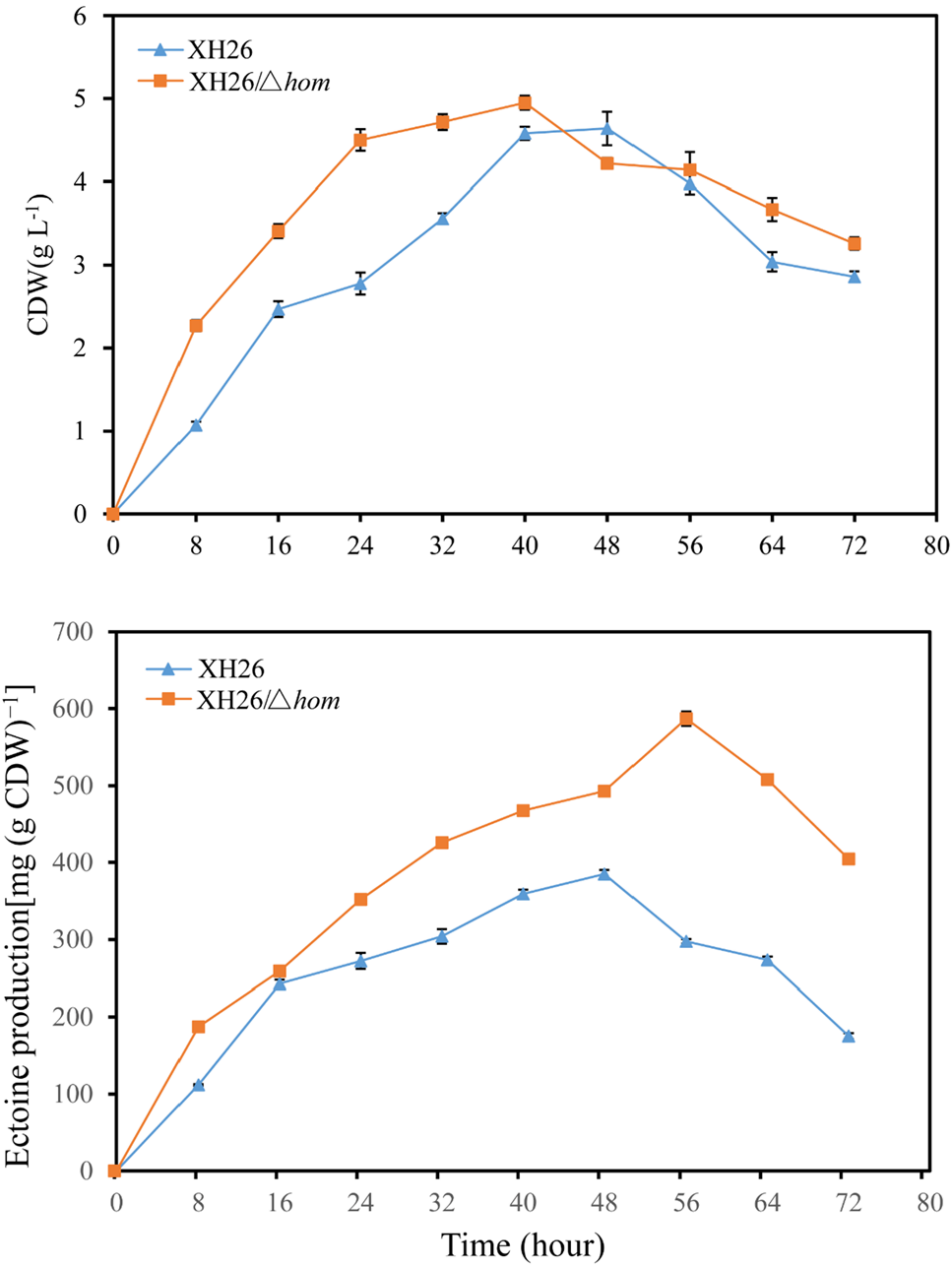
Growth (**a**) and ectoine accumulation (**b**) of the wild-type strain XH26 and the defective strain XH26/*Δhom* under batch fermentation. Each experiment was performed in duplicate.

During the fermentation process for ectoine production, a 0.5% concentration of aspartic acid was employed as a nitrogen source instead of yeast extract in the medium, supplemented with 5 g L^−1^ glucose. In addition, biotin was added to the fermentation medium to facilitate bacterial growth and ectoine production. The growth and ectoine production of wild type strain and XH26/Δ*hom* were compared after 72 h of fermentation in batch (Fig. 5). The results revealed that both strains exhibited a significant increase in growth and ectoine yield, with the defective strain XH26/*Δhom* exhibiting higher growth and ectoine yield than the wild-type XH26 strain. The highest ectoine yield of 587.09 mg (g CDW)^−1^ was obtained by the defective strain XH26/*Δhom* at 56 h, which was higher than that of the wild type strain XH26 at 48 h. The highest ectoine yield of 385.03 mg (g CDW)^−1^ was obtained at 48 h by the wild-type strain XH26 (Fig. 5b). The maximum biomass (CDW) of XH26/*Δhom* was observed at 40 h (4.95 g L^−1^), while that of the wild-type strain was achieved at 48 h (4.64 g L^−1^) (Fig. 5a). Furthermore, the defective strain XH26/*Δhom* produced ectoine earlier than the wild-type strain, with ectoine already being produced by hour 4. Although the ectoine production of the defective strain was higher than that of the wild-type strain during fermentation, the trend of ectoine accumulation was essentially similar between the two strains.

## Discussion

In most ectoine-producing strains, multi-omic crosstalk analysis of biosynthetic initiation, response and network regulation of associated genes in compatible solute ectoine under salt-adapted conditions requires in-depth study^50^. In this context, the transcriptomic analysis of the salt adaptation mechanism of *H. beimenensis* was identified 16 possible related genes (*spoT*, *prkA*, *mtnN2*, *rsbV*, *lon*, *smpB*, *rfbC*, *rfbP*, *tatB*, *acrR1* and *acA*) involved in salt adaptation, and verified that four genes (*qrA, trkA2, nadA* and *gdhB*) are involved in Na^+^ efflux, K^+^ uptake, H^+^ energy conversion and ectoine biosynthesis, respectively, with important regulatory roles^51^. In the *H. campaniensis* strain XH26, metabolic linkage analysis revealed that the main biosynthetic pathway of ectoine (*lysC* + *asd* + *ectABC*) is directly linked to Asp and aspartate semialdehyde metabolism, and the main metabolic streams are closely linked to the upstream Asn, Glu, Gln and Ala metabolic networks. Furthermore, the carbon/nitrogen metabolic flow of ectoine biosynthesis is closely related to the tricarboxylic acid cycle (succinic acid, ferredoxin and oxaloacetate), which may provide new insights for the optimization of the ectoine synthesis pathway and the design of metabolic pathway integration experiments (Glu → TCA → Asp → ectoine).

For the most part, the principles for constructing engineered strains with high ectoine yields are based on improving the utilisation of carbon/nitrogen sources^48^. The low utilization of carbon/nitrogen sources is primarily reflected in the accumulation of too many intermediate products, the coexistence of multiple products, and inefficient synthesis. The under-utilization of carbon sources, which limits ectoine production, is primarily addressed at present using three approaches: (i) Some studies have aimed to increase the input of carbon sources for ectoine synthesis. For example, ectoine synthesis is enhanced by the overexpression of sugar transporter proteins that increase glucose and xylose uptake^49^. (ii) Other studies have aimed to modify the ectoine synthetic pathway based on analyses of the ectoine metabolic pathway, thereby improving carbon source use. Primary pathway-related goals include increasing the activity of key enzymes in the pathway^50^, disruption of futile cycles 51^51^, removing competing amino acid synthesis pathways 52^52^, and blocking the ectoine catabolic pathway^53^. (iii) Finally, some studies have analyzed the effects of carbon supply on ectoine synthesis, aiming to determine how changes in carbon supply and the optimization of fermentation conditions might improve ectoine production and increase of ectoine demand^54,55^. Thus, with the defective strain XH26/*Δhom* constructed in this study, the synthetic precursor substrate metabolic shunt pathway was effectively removed, improving the availability of carbon/nitrogen sources for ectoine synthesis and increasing ectoine production by 1.47-fold relative to XH26.

In this study, a defective strain XH26/*Δhom* was constructed by knocking out the *hom* gene, which disrupted the shunt of the ectoine synthesis precursor substrate l-aspartate 4-semialdehyde, thereby improving the availability of carbon/nitrogen sources for ectoine synthesis^56–58^. As expected, ectoine fermentation experiments revealed that ectoine synthesis by the gene-deficient strain increased under saline culture conditions (0–3 mol NaCl L^−1^) compared to the wild-type strain. Ectoine yields increased by about 4.23-fold under low salinity (0.5 mol NaCl L^−1^), and ectoine yields was 351.13 mg (g CDW)^−1^ at a salinity of 1.5 mol NaCl L^−1^, much higher than the 239.18 mg (g CDW)^−1^ of the wild-type strain. In the 3-L bioreactor, optimising the batch fermentation parameters of nitrogen source, agitation and aeration. After 72 h fermentation, the maximum ectoine yields of XH26/*Δhom* was 587.09 mg (g CDW)^−1^. In addition, the growth of the defective strain also differed to that of the wild type, possible due to the knockout of the *hom* gene. Most notably, the salinity associated with optimal growth differed between the defective strain and the wild type. We suspect that the wild-type *H. campaniensis* strain XH26 produces less ectoine than is required to equilibrate cellular osmotic pressure. Therefore, the increase in the intracellular ectoine content of defective strain XH26/*Δhom* led to a concomitant increase in the salinity associated with optimal growth.

It is worth noting that glycine synthesis is closely related to the betaine metabolism. Knockout of *hom* blocks the synthesis of glycine from l-aspartate-4-semialdehyde, potentially reducing glycine levels in microorganisms and thereby decreasing betaine synthesis or increasing betaine catabolism. Betaine fermentation experiments revealed that betaine synthesis by the gene-deficient strain decreased under saline culture conditions (0–2.5 mol NaCl L^−1^) compared to the wild-type strain. Betaine yields was 19.98 mg g CDW)^−1^ at a salinity of 1.5 mol NaCl L^−1^, much lower than the 69.58 mg (g CDW)^−1^ of the wild-type strain. *Halomonas* species contain diverse compatible solutes and use various substances to counteract salt stress, some of which are antagonistic towards one another^59,60^. For example, betaine inhibits ectoine synthesis under low salinity conditions. Therefore, knocking out *hom* may also improve ectoine production by reducing betaine concentrations.

## Supplementary Information

Supplementary Information.

## Data Availability

The complete genome sequence has been deposited at GenBank under accession number CP071889 (Bioproject number PRJNA705502, Biosample number SAMN18316568).

## References

[1] Czech L, Poehl S, Hub P, Stoveken N, Bremer E (2018). Tinkering with osmotically controlled transcription allows enhanced production and excretion of ectoine and hydroxyectoine from a microbial cell factory. Appl. Environ. Microbiol..

[2] Galinski EA, Pfeiffer HP, Trüper HG (1985). 1,4,5,6-Tetrahydro-2-methyl-4-pyrimidinecarboxylic acid. A novel cyclic amino acid from halophilic phototrophic bacteria of the genus Ectothiorhodospira. Eur. J. Biochem..

[3] Fenizia S, Thume K, Wirgenings M, Pohnert G (2020). Ectoine from bacterial and algal origin is a compatible solute in microalgae. Mar. Drugs..

[4] Bursy J (2008). Synthesis and uptake of the compatible solutes ectoine and 5-hydroxyectoine by *Streptomyces coelicolor* A3(2) in response to salt and heat stresses. Appl. Environ. Microbiol..

[5] Pastor JM (2010). Ectoines in cell stress protection: Uses and biotechnological production. Biotechnol. Adv..

[6] Meyer S (2017). Ectoine can enhance structural changes in DNA in vitro. Sci. Rep..

[7] Held C, Sadowski G (2016). Compatible solutes: thermodynamic properties relevant for effective protection against osmotic stress. Fluid. Phase. Equilib..

[8] Oren A (2011). Thermodynamic limits to microbial life at high salt concentrations. Environ. Microbiol..

[9] Brands S, Schein P, Castro-Ochoa KF, Galinski EA (2019). Hydroxyl radical scavenging of the compatible solute ectoine generates two N-acetimides. Arch. Biochem. Biophys..

[10] Unfried K (2016). Reduction of neutrophilic lung inflammation by inhalation of the compatible solute ectoine: A randomized trial with elderly individuals. Int. J. Chron. Obstruct. Pulmon. Dis..

[11] Wedeking A (2014). A lipid anchor improves the protective effect of ectoine in inflammation. Curr. Med. Chem..

[12] Bownik A, Stępniewska Z (2016). Ectoine as a promising protective agent in humans and animals. Arh. Hig. Rada. Toksikol..

[13] Boroujeni MB, Nayeri H (2018). *Stabilization* of bovine lactoperoxidase in the presence of ectoine. Food. Chem..

[14] Graf R, Anzali S, Buenger J, Pfluecker F, Driller H (2008). The multifunctional role of ectoine as a natural cell protectant. Clin. Dermatol..

[15] Sauer T, Galinski E (1998). Bacterial milking: A novel bioprocess for production of compatible solutes. Biotechnol. Bioeng..

[16] Kunte HJ, Lentzen G, Galinski E (2014). Industrial production of the cell protectant ectoine: Protection mechanisms, processes, and products. Curr. Biotechnol..

[17] Widderich N (2016). Strangers in the archaeal world: Osmostress-responsive biosynthesis of ectoine and hydroxyectoine by the marine thaumarchaeon *Nitrosopumilus maritimus*. Environ. Microbiol..

[18] Louis P, Galinski EA (1997). Characterization of genes for the biosynthesis of the compatible solute ectoinefrom *Marinococcus halophilus* and osmoregulated expression in *Escherichia coli*. Microbiology.

[19] Ono H (1999). Characterization of biosynthetic enzymes for ectoine as a compatible solute in a moderately *Halophilic eubacterium, Halomonas elongata*. J. Bacteriol..

[20] Richter AA (2020). The architecture of the diaminobutyrate acetyltransferase active site provides mechanistic insight into the biosynthesis of the chemical chaperone ectoine. J. Biol. Chem..

[21] Eilert E, Kranz A, Hollenberg CP, Piontek M, Suckow MJ (2013). Synthesis and release of the bacterial compatible solute 5-hydroxyectoine in *Hansenula polymorpha*. J. Biotechnol..

[22] Schubert T, Maskow T, Benndorf D, Harms H, Breuer U (2007). Continuous synthesis and excretion of the compatible solute ectoine by a transgenic, nonhalophilic bacterium. Appl. Environ. Microbiol..

[23] Perez-Garcia F, Ziert C, Risse JM, Wendisch VF (2017). Improved fermentative production of the compatible solute ectoine by *Corynebacterium glutamicum* from glucose and alternative carbon sources. J. Biotechnol..

[24] Parwata IP, Wahyuningrum D, Suhandono S, Hertadi RJ (2019). Heterologous ectoine production in *Escherichia coli*: Optimization using response surface methodology. Int. J. Microbiol..

[25] Li S (2021). Promoter engineering for high ectoine production in a lower saline medium by *Halomonas hydrothermalis* Y2. Biotechnol. Lett..

[26] Ning Y (2016). Pathway construction and metabolic engineering for fermentative production of ectoine in *Escherichia coli*. Metab. Eng..

[27] Xie, X. X. *et al.* Genetically engineered bacteria using xylose to induce ectoine production and its application: CHN, CN106754603A. 2017-05-31.

[28] Chen J (2020). Metabolic pathway construction and optimization of *Escherichia coli* for high-level ectoine production. Curr. Microbiol..

[29] Zhang T (2020). Study of osmoadaptation mechanisms of halophilic *Halomonas alkaliphila* XH26 under salt stress by transcriptome and ectoine analysis. Extremophiles.

[30] Schwibbert K (2010). A blueprint of ectoine metabolism from the genome of the industrial producer *Halomonas elongata* DSM 2581T. Environ. Microbiol..

[31] Göller K (1998). Construction and characterization of an NaCl-sensitive mutant of *Halomonas elongata* impaired in ectoine biosynthesis. FEMS. Microbiol. Lett..

[32] Ermert AL, Nogué F, Stahl F, Gans T, Hughes J (2019). CRISPR/Cas9-mediated knockout of Physcomitrella patens Phytochromes. Methods. Mol. Biol..

[33] Sahoo N (2020). CRISPR-Cas9 genome editing in human cell lines with donor vector made by gibson assembly. Methods. Mol. Biol..

[34] Hou XW, Tong HY, He ZH (2021). Alternative seamless cloning strategies in fusing gene fragments based on overlap-PCR. Mol. Biotechnol..

[35] Zhang Y (2007). A novel PEGylation of chitosan nanoparticles for gene delivery. Biotechnol. Appl. Biochem..

[36] Cheng C, Jia JL, Ran SY (2015). Polyethylene glycol and divalent salt-induced DNA reentrant condensation revealed by single molecule measurements. Soft Matter.

[37] Zhang Z (2023). Effects of adipose derived stem cells pretreated with resveratrol on sciatic nerve regeneration in rats. Sci. Rep..

[38] Chen R (2017). Optimization of the extraction and purification of the compatible solute ectoine from *Halomonas elongate* in the laboratory experiment of a commercial production project. World. J. Microbiol. Biotechnol..

[39] Vandrich J, Pfeiffer F, Alfaro-Espinoza G, Kunte HJ (2020). Contribution of mechanosensitive channels to osmoadaptation and ectoine excretion in *Halomonas elongata*. Extremophiles.

[40] Cantera S (2020). A systematic comparison of ectoine production from upgraded biogas using *Methylomicrobium alcaliphilum* and a mixed haloalkaliphilic consortium. Waste. Manag..

[41] Pérez-García F, Ziert C, Risse JM, Wendisch VF (2017). Improved fermentative production of the compatible solute ectoine by *Corynebacterium glutamicu*m from glucose and alternative carbon sources. J. Biotechnol..

[42] Chen PW (2020). Exploring the additive bio-agent impacts upon ectoine production by *Halomonas salina* DSM5928^T^ using corn steep liquor and soybean hydrolysate as nutrient supplement. J. Biosci. Bioeng..

[43] Dong Y (2021). Enhancing ectoine production by recombinant *Escherichia coli* through step-wise fermentation optimization strategy based on kinetic analysis. Bioprocess. Biosyst. Eng..

[44] Zhao Q (2019). High ectoine production by an engineered *Halomonas hydrothermalis* Y2 in a reduced salinity medium. Microb. Cell. Fact..

[45] Kanehisa M, Goto S (2000). KEGG: Kyoto encyclopedia of genes and genomes. Nucleic. Acids. Res..

[46] Kanehisa M (2019). Toward understanding the origin and evolution of cellular organisms. Protein. Sci..

[47] Kanehisa M, Furumichi M, Sato Y, Kawashima M, Ishiguro-Watanabe M (2023). KEGG for taxonomy-based analysis of pathways and genomes. Nucleic. Acids. Res..

[48] Duan Y (2022). Systematic metabolic engineering for the production of azaphilones in *Monascus purpureus* HJ11. J. Agric. Food. Chem..

[49] Göller K, Ofer A, Galinski EA (1998). Construction and characterization of an NaCl-sensitive mutant of *Halomonas elongata* impaired in ectoine biosynthesis. FEMS. Microbiol. Lett..

[50] Reshetnikov AS (2020). Ectoine degradation pathway in halotolerant methylotrophs. PLoS ONE.

[51] Chen YH, Lu CW, Shyu YT, Lin SS (2017). Revealing the saline adaptation strategies of the Halophilic Bacterium *Halomonas beimenensis* through high-throughput omics and transposon mutagenesis approaches. Sci. Rep..

[52] Tanimura K, Matsumoto T, Nakayama H, Tanaka T, Kondo A (2016). Improvement of ectoine productivity by using sugar transporter-overexpressing *Halomonas elongata*. Enzyme. Microb. Technol..

[53] Stöveken N (2011). A specialized aspartokinase enhances the biosynthesis of the osmoprotectants ectoine and hydroxyectoine in *Pseudomonas stutzeri* A1501. J. Bacteriol..

[54] Jiang A (2022). High-yield ectoine production in engineered *Corynebacterium glutamicum* by fine metabolic regulation via plug-in repressor library. Bioresour. Technol..

[55] Cho S (2022). Enhanced production of ectoine from methane using metabolically engineered *Methylomicrobium alcaliphilum* 20Z. Biotechnol. Biofuels. Bioprod..

[56] Kunte HJ, Lentzen G, Galinski EA (2022). Industrial production of the cell protectant ectoine: Protection mechanisms, processes, and products. Curr. Biotechnol..

[57] Gießelmann G (2019). Metabolic engineering of *Corynebacterium glutamicum* for high-level ectoine production: Design, combinatorial assembly, and implementation of a transcriptionally balanced heterologous ectoine pathway. Biotechnol. J..

[58] Hobmeier K (2022). Metabolic engineering of *Halomonas elongata*: Ectoine secretion is increased by demand and supply driven approaches. Front. Microbiol..

[59] Omara A (2020). Optimizing ectoine biosynthesis using response surface methodology and osmoprotectant applications. Biotechnol. Lett..

[60] Kushwaha B (2019). Betaine accumulation suppresses the de-novo synthesis of ectoine at a low osmotic concentration in *Halomonas* sp SBS 10, a bacterium with broad salinity tolerance. Mol. Biol. Rep..

