# Supplementary material for: Metabolic engineering of Halomonas campaniensis strain XH26 to remove competing pathways to enhance ectoine production
Source: Sci Rep. 2023 Jun 15;13:9732. doi: 10.1038/s41598-023-36975-8 (PMC10272175; doi:10.1038/s41598-023-36975-8)
Supplement: Supplementary file 1 — Supplementary Information. [file 41598_2023_36975_MOESM1_ESM.docx]

Metabolic engineering of *Halomonas campaniensis* strain XH26 to remove competing pathways to enhance ectoine production

Zhiwan Shu · Xin Zhang · Rong Wang · Jiangwa Xing · Yongzhen Li · Derui Zhu· Guoping Shen*

*Research Center of Basic Medical Science, Medical College of Qinghai University, Xining 810016,* ***,*** *People’s Republic of China*

*Corresponding author:

Prof. Guoping Shen

Research Center of Basic Medical Science, Medical College of Qinghai University, Xining 810016, People’s Republic of China

E-mail: sgpkkll@126.com

The location of *hom* gene in the XH26 genome: 533059-534405. The length of *hom* gene DNA sequence is 1347bp.

ATGGGTGCCCATCACGAAAGGGGAAGTATTTTGAAACCGGTAAGAGTAGGCATTTGTGGGTTAGGTACAGTCGGTGGCGGTACATTTAACGTCTTAACACGTAACGCTGATGACATTAGCCGTCGTGCTGGCCGCCCGATTGTGATTGAGCAGGTTGCCCACCGCAGTATTCACCCTGACTGTGATATTACCGGCATTAATGCGACTTCTGACGTGTTTGAGGTGGCTAATAACCCTAACGTGGATGTGCTGGTAGAGCTGATTGGTGGCTACGATATCGCCCGCGAGCTGGTGCTAACGGCGATTGAAAACGGCAAACACGTTGTGACTGCCAACAAGGCATTGATTGCTGTTCACGGTAATGAAATTTTCCGCGCCGCGCATAAGAAAGGCGTCATTGTTGCCTTTGAGGCGGCTGTGGCGGGCGGTATTCCGGTCATCAAATCGCTACGCGAAGGCCTTGGTGCTAACCGTATCGAGTGGGTAGCGGGCATCATAAACGGCACCGGTAACTACATTCTCACCCATATGCGTGATGAAGGTCGCGCGTTTGAAGACGTGTTGGCCGAAGCCCAAGCGCTAGGTTACGCGGAGTCTGACCCCACTTTCGACGTGGAAGGTATCGACGCTGCCCATAAATTGACCATCCTGGCGTCAATTGCCTATGGCGTGCCGCTGCAGTTTGATAAAGCCTTCACCGAAGGTATTTCTCGCATTACTGCAGAAGACGTCGAGCAGGCCGATAACCTGGGTTACGTGATTAAGCATCTGGGCATCTCTAAACGGACTGATCAAGGGCTAGAGCTCCGAGTTCACCCGACGTTGATTCCTAAAGAGCGCCTGCTGGCGAATGTGCACGGCGTTAAAAATGCGATTGCTGTGATGGGCGATGCGGTTGGTCCAACACTTTACTACGGCGCTGGCGCCGGTGCTGAGCCGACAGCGTCTGCCGTCGTAGCCGACCTGCTGGATGTTGCCCGTGATATCGCCACGGATCACCACTACCGGGTGCCTTACCTAGCGTTTAGCGGCATCGACGATGATGCCAGTCAACTGCCTATTATGCCGATGGAAGATATTACCACAGCGTATTATCTGCGTCTGTTGGCGGTGGATCGCCCGGGTGTGTTGGCGCGTGTTGCCACCATTCTGGCCGAGCAGGGCATCTCTATTGAAGCGCTGATCCAGAAAGAGGCTACTGAAGGTGAGTTGGTGCCAATCATTTTGCTGACGCATCGCACAAAAGAGAAACAGATGAACGATGCTATCCGTGAAATCGAGTCCATGGCGGATATTGCTGGGCCGGTTACCCGTATTCGCGTTGAAAGTCTGAGCGAAGGGGAGTAA

The *hom* gene with its upstream and downstream sequences.

CTTGTCGCCTCACACGTTACGCCACGCATTTGCGACTCATTTATTGAATCATGGTGCTAATTTGCGGGTCGTACAGCTGTTGTTAGGTCATAGCGACCTATCTACAACGCAAATTTATACGCATGTTGCCCAGGCGCGCCTGGAGCAATTGCATGCCGAACACCATCCGCGAGGTTGAAGATACAATGCATCAACGTATGTCTTTAGTTGCTAAATCCTGTGTTCCCTGGCTGGCGCTTGCGGGGTGTTTACTGCCTGCTGTTGCCAGTGCTGACTCCGTTTCCGAGTATCTGACGGAGAACTTGCAAGTTAACGGCCAGTCAATGCCGGTGGAGCAGGTAAACGCAACGCCAATGGACGGGGTGTACCACGTTGTTCTGGAAAGTGGTGAATCTTTTTACTCCAATGCCGATGGTAGCCACTTTTTGGTAGGGGACCTCTATCAAAACGCGGATAATGGTTTGGTTAACTTAACCGAGCAGGCGCGTAACCAAGAAAGGGCTACAGTGCTTGCTGCCATTCCAGCGAGCGAGCGAGTCGTTTTTCAAGGTGTGGGTGAGCCGAAAGCTACGGTAATTGTGTTTACGGATCCTACCTGCCCATACTGCGAGCGGCTACATGAAACGGTGCCTGAGCTTAACGAGCGCGGCATTGCAGTGCACTATATGGCCTTTCCACGCGCAGGCATGGGCAGTGGTGCTGCGACGACGCTTGAGCAAGTGTGGTGTTCGGATAACCGTAGCGAGGCCATGACTCAAGCTAAACAAGGCCAAACGCTTGCTGCATCGGCAAACTGCGATAACCCCGTTGCCGATCAGTACGAATTAGGTAAGGCTGTGGGCGTGCAGGGTACTCCTGCGATTGTGTTGCCCGACGGGAAAATGGTGCCAGGGTTTGTACCCCCCGACCGGCTCGTTAGCATGTTAGGCTTGGAAGACGAATAACGTCACGTGCAGTGACGAACGATAGCGCTGAGACACTGATTTAAGAGGCGGCACCTATGGGTGCCCATCACGAAAGGGGAAGTATTTTGAAACCGGTAAGAGTAGGCATTTGTGGGTTAGGTACAGTCGGTGGCGGTACATTTAACGTCTTAACACGTAACGCTGATGACATTAGCCGTCGTGCTGGCCGCCCGATTGTGATTGAGCAGGTTGCCCACCGCAGTATTCACCCTGACTGTGATATTACCGGCATTAATGCGACTTCTGACGTGTTTGAGGTGGCTAATAACCCTAACGTGGATGTGCTGGTAGAGCTGATTGGTGGCTACGATATCGCCCGCGAGCTGGTGCTAACGGCGATTGAAAACGGCAAACACGTTGTGACTGCCAACAAGGCATTGATTGCTGTTCACGGTAATGAAATTTTCCGCGCCGCGCATAAGAAAGGCGTCATTGTTGCCTTTGAGGCGGCTGTGGCGGGCGGTATTCCGGTCATCAAATCGCTACGCGAAGGCCTTGGTGCTAACCGTATCGAGTGGGTAGCGGGCATCATAAACGGCACCGGTAACTACATTCTCACCCATATGCGTGATGAAGGTCGCGCGTTTGAAGACGTGTTGGCCGAAGCCCAAGCGCTAGGTTACGCGGAGTCTGACCCCACTTTCGACGTGGAAGGTATCGACGCTGCCCATAAATTGACCATCCTGGCGTCAATTGCCTATGGCGTGCCGCTGCAGTTTGATAAAGCCTTCACCGAAGGTATTTCTCGCATTACTGCAGAAGACGTCGAGCAGGCCGATAACCTGGGTTACGTGATTAAGCATCTGGGCATCTCTAAACGGACTGATCAAGGGCTAGAGCTCCGAGTTCACCCGACGTTGATTCCTAAAGAGCGCCTGCTGGCGAATGTGCACGGCGTTAAAAATGCGATTGCTGTGATGGGCGATGCGGTTGGTCCAACACTTTACTACGGCGCTGGCGCCGGTGCTGAGCCGACAGCGTCTGCCGTCGTAGCCGACCTGCTGGATGTTGCCCGTGATATCGCCACGGATCACCACTACCGGGTGCCTTACCTAGCGTTTAGCGGCATCGACGATGATGCCAGTCAACTGCCTATTATGCCGATGGAAGATATTACCACAGCGTATTATCTGCGTCTGTTGGCGGTGGATCGCCCGGGTGTGTTGGCGCGTGTTGCCACCATTCTGGCCGAGCAGGGCATCTCTATTGAAGCGCTGATCCAGAAAGAGGCTACTGAAGGTGAGTTGGTGCCAATCATTTTGCTGACGCATCGCACAAAAGAGAAACAGATGAACGATGCTATCCGTGAAATCGAGTCCATGGCGGATATTGCTGGGCCGGTTACCCGTATTCGCGTTGAAAGTCTGAGCGAAGGGGAGTAACTCATGCGTTATATCAGCACGCGTGGCCAAGCGCCCGCGCTCTCCTTTGAAGAGGTTGTGCTAACCGGAATGGCCAGCGACGGTGGACTTTATGTGCCGGAAACGCTGCCCGAGTTTTCTAAGGAAGAGCTGGCCAGCATGGCTGGCCTTTCCTACGCCGAGATTGCTTTTCGGGTCATGAAGCCGTTTGTAAATGGTGAAATTGACGACGATACCTTCCGTCGCTTGGTGACAGAAGCTTACGCCACGTTCAATCATGACGCCGTTGTGCCGTTAAAGCAGTTGAATGCCAATCACTTTCTGCTAGAGCAGTTCCATGGCCCAACGCTGGCGTTTAAAGATGTCGCGCTGCAGCTGCTAGGGCGGCTACTGGATCATTTCCTGAAAAAGCGTGGTGAACGTGCCGTCATTATGGGCGCGACCTCTGGGGATACCGGTTCCGCGGCTATTGAAGGCTGTCGCCACTGCGATAACCTCGATATTTTTATTCTCCACCCGCATAACCGCGTGTCTGAAGTGCAGCGCCGCCAGATGACGTCGGTGCTCGCCAACAACGTCTTCAATATTGCTATCGAAGGCAATTTTGATGATGCCCAGGCAATGGTTAAGGCGAGCTTTGCTAATCAGGATTTCCTGAATGGCACGCGCTTAGTGGCGGTGAACTCGATCAACTGGGCGCGCATTATGGCGCAGATCGTGTACTACATCGCCGCTGGTGTTGCATTGGGCGCGCCTCAGCGAGAAGTGAGCTTCTGCGTTCCGTCGGCTAACTTCGGTAACGTCTTCGCAGGCTACATGGCGTTTAAGATGGGGCTGCCGGTGAAGCAGTTCATTATTGCCACTAATGCCAACGACATTCTGCATCGCACGCTTGCCGCCAATGATTTCTCCAAGAAAGAGTTGGCAGCGACCCTCGCGCCGTCAATGGACATCGTTGTTTCGTCAAACTTTGAGCGTTTGCTGTTTGATGCATACGACCGCGATGGTGCCGCTGTGGCAG

#

# Construction and transcription of the sgRNA vector

The pUC57-sgRNA vector (2,879 bp) was successfully constructed by ligating pUC57 (2,710 bp) to the sgRNA (169 bp). DNA sequencing showed that the vector plasmids had 100% nucleotide sequence identity. sgRNA concentration was 1,902 ng sgRNA µL−1, and sgRNA purity was consistent with experimental requirements (i.e., an OD260/280 value of 1.91). Furthermore, the sgRNA electrophoresis band size was consistent with the expected results (Fig. 1). These results demonstrated that the pUC57-sgRNA plasmid vector had been successfully constructed.


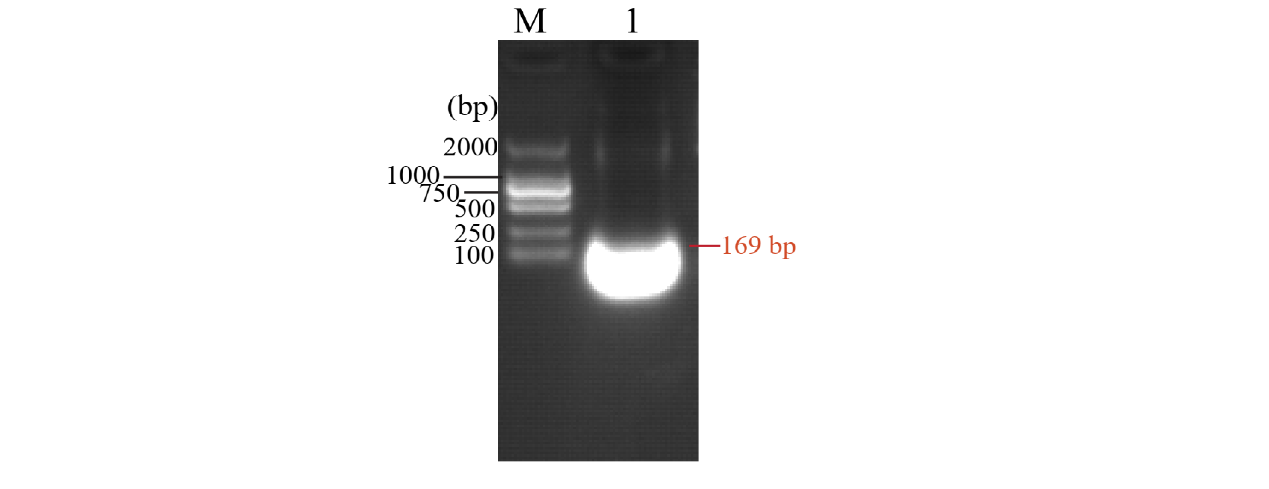


**Fig. 1** Gel electrophoresis results showing sgRNA expression. Lane M: DNA marker, lane 1: sgRNA. The electrophoretic gels and blots in the manuscript have been cropped to improve the clarity and brevity of the presentation.

**Acquisition and connection of the left and right arms of the donor vector**

The left and right arms of the donor vector were obtained via PCR amplification with genomic DNA as the template, and the PCR products were verified using gel electrophoresis and sequencing (Fig. 2a). The sequences of the left and right arms of the donor vector obtained by PCR amplification were 972 bp and 1,003 bp, respectively, and both sequences were identical to the reference genomic sequence. The left and right arms of the donor vector were ligated by overlap PCR using pMD19-T-L and pMD19-T-R as the templates (Fig. 2b), resulting in a product band size of 1,975 bp, which was consistent with the expected results. DNA sequencing confirmed the expected sequence and showed that the sequences were free of mutations and base changes at sequence junctions. Bacterial liquid PCR also confirmed that the product band size was consistent with the expected results (Fig. 2c).


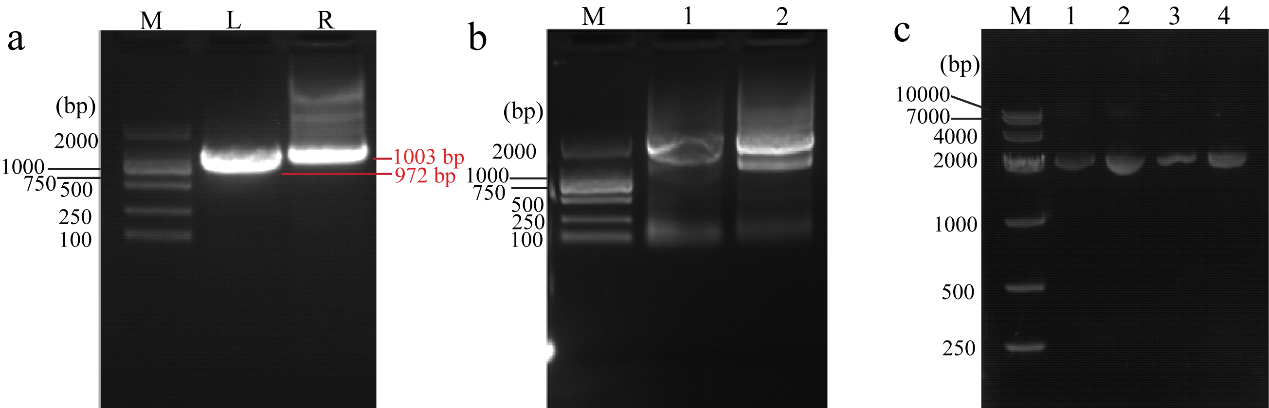


**Fig. 2** PCR amplification and DNA sequencing verification of left and right arm donor vectors. **a**, PCR amplification of the left and right arm of the donor vector. Lane M: DNA marker, lane L: left-arm amplificons, lane R: right-arm amplicons. **b**, Overlap PCR amplification of the left and right arms of the ligated donor vector. Lane M: DNA marker, lanes 1 and 2: pMD19-T-L+R. **c,** Validation of bacterial liquid PCR. Lane M: DNA marker, lanes 1–4: pMD19-T-L+R. The electrophoretic gels and blots in the manuscript have been cropped to improve the clarity and brevity of the presentation.

**Defective strain screening and validation**

Defective strains were obtained by transforming the plasmids pMD19-T-L+R, pUC57-sgRNA, and pwtCas9-bacteria into XH26 competent cells. Cas9 protein expression was induced in the recombinants via exposure to ampicillin and tetracycline, and protein expression was verified using SDS-PAGE analysis (Fig. 3). After induction with ampicillin and tetracycline, Cas9 protein expression increased significantly, and the size of the expressed protein was consistent with the expectations (130 kDa). This indicated that the pwtCas9-bacteria plasmid was successfully transformed into the competent XH26 cells, and that the Cas9 protein was appropriately expressed. Successful transformation was confirmed by screening with agar plates containing ampicillin and tetracycline. A total of 19 potentially defective strains were selected for subsequent analysis (Fig. 4). PCR validation of the defective strains revealed that the samples in lanes 6 and 19 of the electrophoresis gel produced substantially smaller bands than samples in the other lanes, suggesting that these samples corresponded to defective strains. Consistent with this, DNA sequencing of the PCR products revealed that *hom* was knocked out in the cultures represented by lanes 6 and 19. Thus, these cultures contained the defective strains.


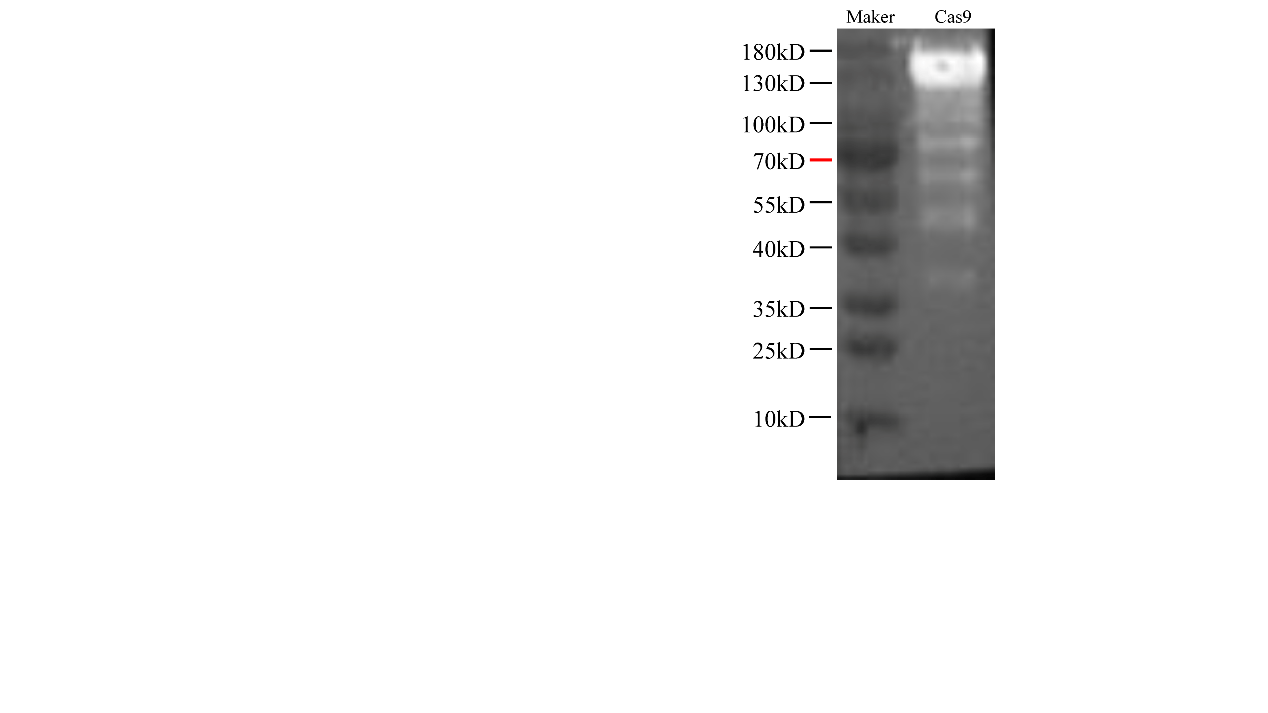


**Fig. 3** SDS-PAGE analysis of Cas9 protein expression. Lane Cas9: XH26 expression after induction by ampicillin and tetracycline. The electrophoretic gels and blots in the manuscript have been cropped to improve the clarity and brevity of the presentation.


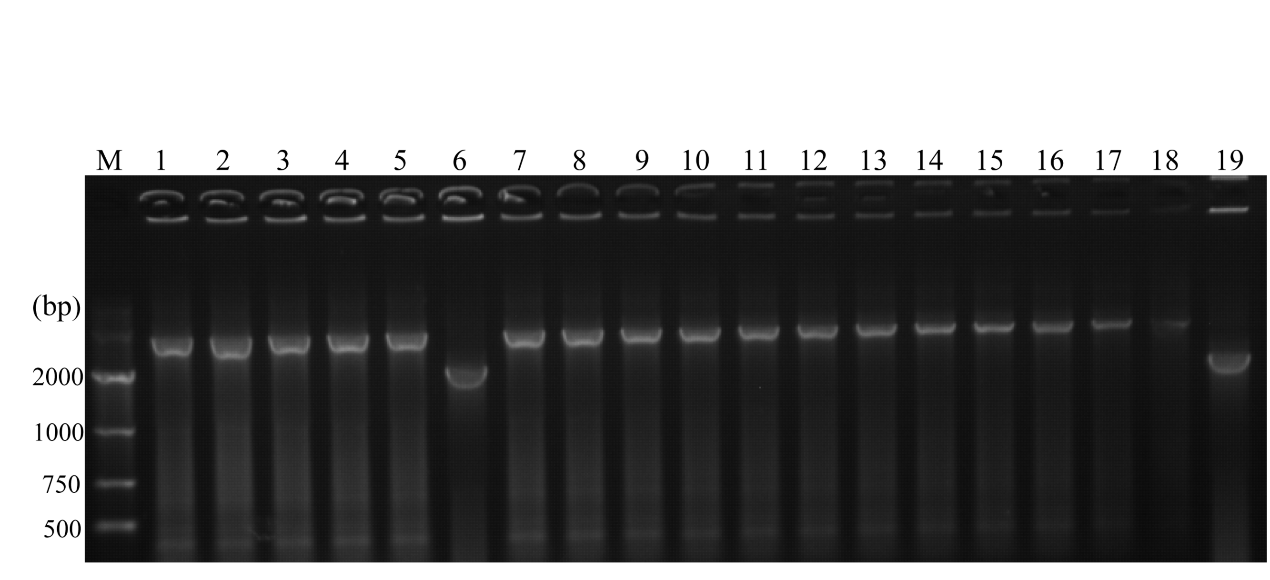


**Fig. 4** PCR amplification screening and DNA sequencing validation of the XH26/*Δhom* defective strain. **a**, Strain XH26/*Δhom* lacked *hom*, as demonstrated by PCR amplification. Lane M: DNA marker; lane 1: wild-type strain XH26; lanes 2–19: monoclonal strains. The electrophoretic gels and blots in the manuscript have been cropped to improve the clarity and brevity of the presentation.


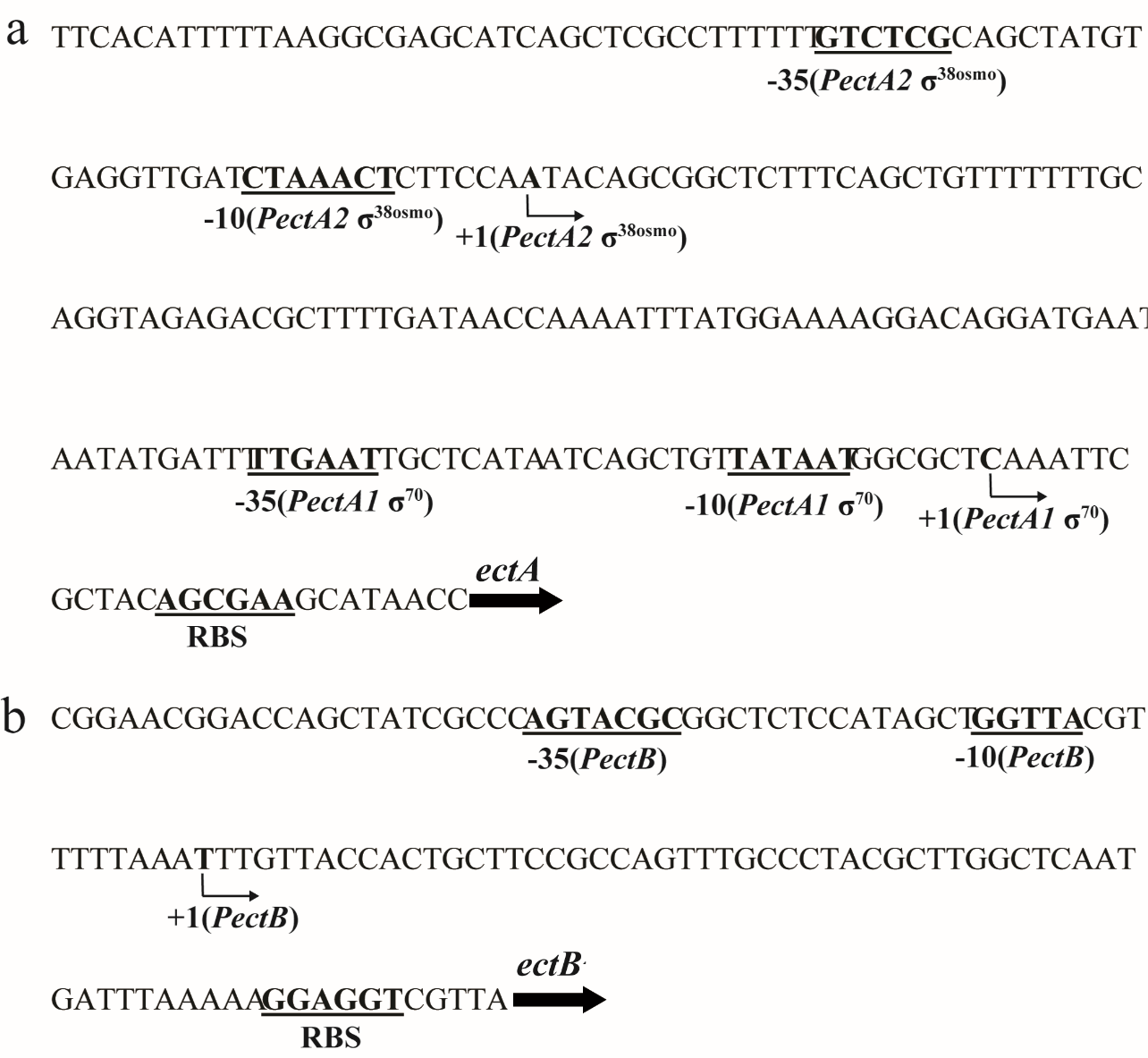


**Fig. 5** Schematic diagram promoter regions upstream of *ectA* (a) and *ectB* (b) in H. campaniensis strain XH26. The transcription start sites (+1) was written in bold. The -35 and -10 sequences of the putative promoters are predicted by sequence analysis and shown in bold

**
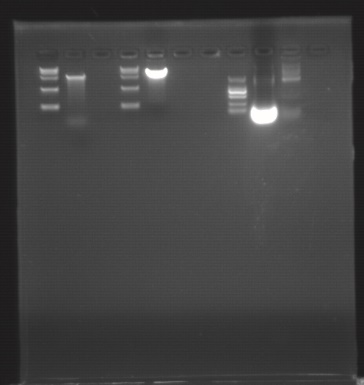
**

Original image of the full-length blot in Fig. 1


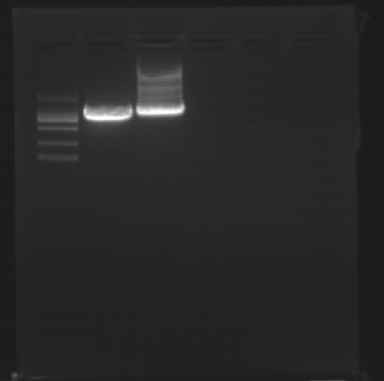


Original image of the full-length blot in Fig. 2a


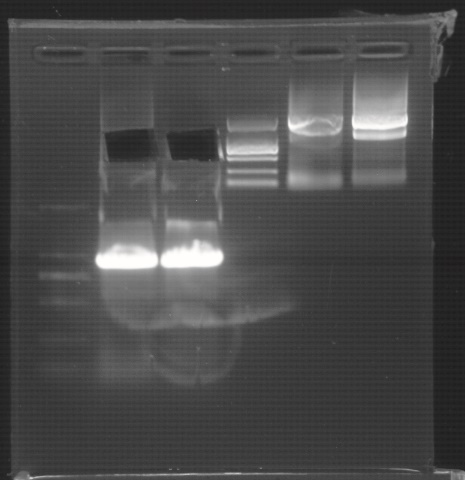


Original image of the full-length blot in Fig. 2b


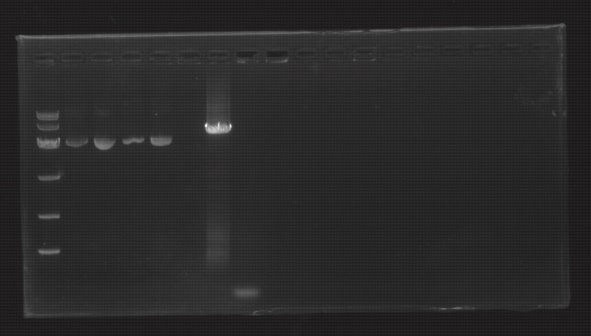


Original image of the full-length blot in Fig. 2c


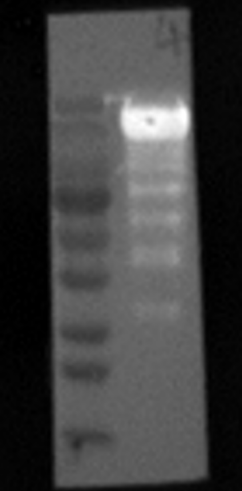


Original image of the full-length blot in Fig. 3


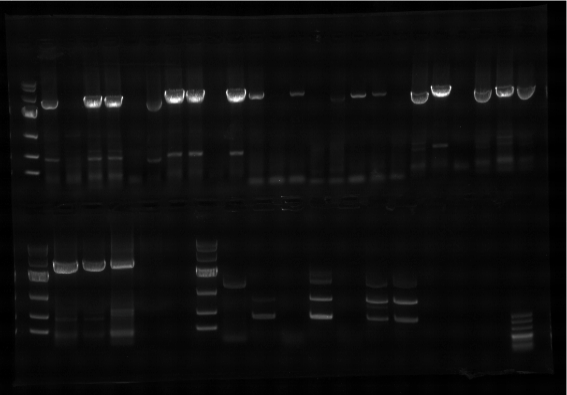

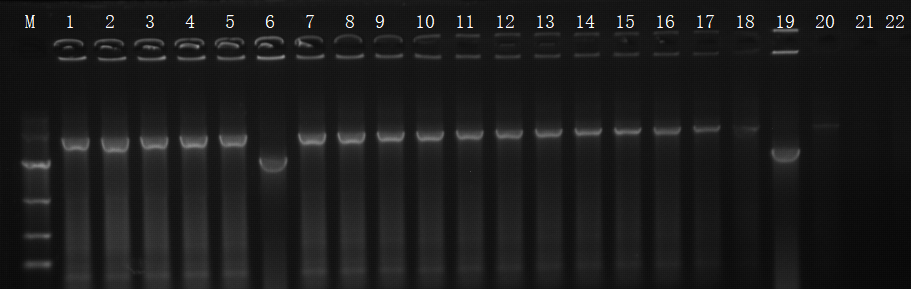


Original image of the full-length blot in Fig. 4
